# Modeling and Optimizing *in vitro* Sterilization of Chrysanthemum via Multilayer Perceptron-Non-dominated Sorting Genetic Algorithm-II (MLP-NSGAII)

**DOI:** 10.3389/fpls.2019.00282

**Published:** 2019-03-14

**Authors:** Mohsen Hesami, Roohangiz Naderi, Masoud Tohidfar

**Affiliations:** ^1^Department of Horticultural Science, Faculty of Agriculture, University of Tehran, Karaj, Iran; ^2^Department of Plant Biotechnology, Faculty of Life Science and Biotechnology, Shahid Beheshti University, Tehran, Iran

**Keywords:** artificial intelligence, data-driven model, *in vitro* culture, optimization algorithm, sensitivity analysis

## Abstract

*In vitro* sterilization is a primary step of plant tissue culture which the ultimate results of *in vitro* culture are directly depended on the efficiency of the sterilization. Artificial intelligence models in a combination of optimization algorithms could be beneficial computational approaches for modeling and optimizing *in vitro* culture. The aim of this study was modeling and optimizing *in vitro* sterilization of chrysanthemum, as a case study, through Multilayer Perceptron- Non-dominated Sorting Genetic Algorithm-II (MLP-NSGAII). MLP was used for modeling two outputs including contamination frequency (CF), and explant viability (EV) based on seven variables including HgCl_2_, Ca(ClO)_2_, Nano-silver, H_2_O_2_, NaOCl, AgNO_3_, and immersion times. Subsequently, models were linked to NSGAII for optimizing the process, and the importance of each input was evaluated by sensitivity analysis. Results showed all of the R^2^ of training and testing data were over 94%. According to MLP-NSGAII, optimal CF (0%), and EV (99.98%) can be obtained from 1.62% NaOCl at 13.96 min immersion time. The results of sensitivity analysis showed that CF and EV were more sensitive to immersion time and less sensitive to AgNO_3_. Subsequently, the performance of predicted and optimized sterilants × immersion times combination were tested, and results indicated that the differences between the MLP predicted and validation data were negligible. Generally, MLP-NSGAII as a powerful methodology may pave the way for establishing new computational strategies in plant tissue culture.

## Introduction

Chrysanthemum is known as one of the well-known multi-disciplinary species used as a pot, cut, and herbaceous plant worldwide (Da Silva and Kulus, 2014). In general, breeders have made extensive use of the conventional breeding approaches to evaluate desirable traits but still need some techniques due to lack of cross-incompatibility and gene pool resources (Noda et al., 2017). Meanwhile, application of biotechnology in the breeding area could pave the way of evaluating desirable traits by making *in vitro* regeneration procedures more efficient such as the production of numerous high quality plants in a relatively short time (Naing et al., 2013; Hesami and Daneshvar, 2016, 2018; Hesami et al., 2018a,b). However, contamination during *in vitro* regeneration procedures is one of the greatest problems that can act as a barrier for making this technique more efficient (Da Silva and Kulus, 2014; Hesami et al., 2017a).

Contamination in plant tissue cultures can be produced by various micro-arthropods (mites, trips, and their vectors), microorganisms (filamentous fungi, yeasts, bacteria), viruses, and viroids (Altan et al., 2010; Da Silva et al., 2016a; Hesami et al., 2018c). Therefore, sterilization step is of high paramount during establishing and maintaining plants in *in vitro* cultures. Sterilization of equipment should not be a serious deal in a modern and well-equipped laboratory due to the frequent use of novel microwave-based autoclaves (Da Silva et al., 2016a). However, serious problems are made during the disinfection of biological material (e.g., initial explant) that need more attention and time (Hesami et al., 2018c). In addition, tissues can be a significant potential of hosting different microorganisms thus adequate and proper sterilization treatments require prior to *in vitro* culture initiation (Mihaljević et al., 2013). The efficiency of disinfection can be influenced by numerous factors such as the size, age, and type of the explant, the conditions of cultivation and physiological state of the stock plant, time and temperature of exposure, and the type of disinfectant and its concentration (Da Silva et al., 2016b). Besides, these mentioned factors can exert a negative impact on the survival and regeneration potential of candidate explant which is imperative for maximizing the efficient transformation system of plant tissue cultures (Da Silva et al., 2016a; Hesami et al., 2018c). There are several types of disinfectants including hydrogen peroxide (H_2_O_2_), mercury II chloride (HgCl_2_), Nano-silver (NS), calcium hypochlorite [Ca(ClO)_2_], silver nitrate (AgNO_3_), sodium hypochlorite (NaOCl), and chemotherapeutics (fungicides, antibiotics) that can be used in different contamination levels and conditions (Mihaljević et al., 2013; Nongalleima et al., 2014). Base on different reports (Nongalleima et al., 2014; Da Silva et al., 2016a; Hesami et al., 2018c), the longer treatment with more concentrated disinfectants used, the better asepsis results will achieve. However, there is a negative correlation with the high concentration of the disinfectants and the rate of explant viability (Nongalleima et al., 2014; Da Silva et al., 2016a; Hesami et al., 2018c). Therefore, the exposure time and concentration of disinfection agents should be adjusted based on various types, age, and species of explants to achieve the best results during *in vitro* sterilization.

The necessity of using appropriate methods for modeling and optimizing possible prediction of *in vitro* culture and growth kinetics can be explained by several non-linear biological processes that easily detected in plant tissue culture (Arab et al., 2016, 2018; Nezami-Alanagh et al., 2018). The demerit point of using conventional analytical techniques based on mathematical models would be obvious due to their unconformity of the non-idealities of *in vitro* culture process (Gago et al., 2010a,b, 2011, 2014; Arab et al., 2016, 2018; Nezami-Alanagh et al., 2018; Niazian et al., 2018). However, Artificial Neural Network (ANN) based modeling methods have to be more useful and flexible in dealing with possible non-linear relationships in *in vitro* culture (Jamshidi et al., 2016). There are different types of ANNs such as Generalized Regression Neural Network (GRNN), Multilayer Perceptron (MLP), Radial basis function (RBF), and Probabilistic Neural Network (PNN) (Araghinejad et al., 2017) that have no dependency on any previous knowledge regarding the construction or inter-relationships between input and output signals. Therefore, the usage of these kinds of models such as ANN would be useful in modeling and optimizing *in vitro* procedures during plant tissue culture (Arab et al., 2016, 2018; Jamshidi et al., 2016). Previous studies pointed out the effectiveness of ANN models over conventional regression methods such as forward, backward or stepwise to make accurate modeling and predicting in plant tissue culture (Gago et al., 2010a,b, 2011, 2014; Arab et al., 2016, 2018; Hesami et al., 2017b; Nezami-Alanagh et al., 2018). However, there is a lack of extensive studies regarding the effectiveness of ANNs in order to assess the best complicated and non-linear relationships among *in vitro* sterilization. Based on our knowledge, this study is the first report of *in vitro* culture modeling of chrysanthemum.

The performance of the plant tissue culture systems in optimization problems can be evaluated by multi-objective functions. There are many trials and errors to optimize the inputs. Recent studies have used a genetic algorithm (GA) to reduce computational volumes (Arab et al., 2016, 2018; Nezami-Alanagh et al., 2017). GA, as the best-known optimization algorithm, causes to achieve the optimal solutions with minimal computing. On the other hand, plant tissue culture problems have to satisfy various objective functions by considering different constraints. However, GA as a single-objective algorithm cannot optimize multi-objective functions, simultaneously (Bozorg-Haddad et al., 2016a; Hosseini-Moghari et al., 2017). Therefore, the multi-objective algorithm has been required for optimization of outputs (Bozorg-Haddad et al., 2016b; Li and Wong, 2018a). The importance of using multi-objective evolutionary optimization for different areas of plant science was emphasized in previous studies (Li and Wong, 2017, 2018b; Li et al., 2018a). Classical optimization methods consist of multi-criterion decision-making methods, providing the converting model of the multi-objective optimization problem to a single-objective optimization problem by emphasizing one particular Pareto-optimal solution at a time. Considering this method for multiple solutions, it has to be applied so many times for finding various solutions at each simulation run (Bozorg-Haddad et al., 2016a; Li et al., 2018b). The Non-dominated Sorting Genetic Algorithm-II (NSGA-II) has been known as the first evolutionary multi-objective optimization algorithms try to find the solution domain for discovering Pareto-optimal solutions within a multi-objective centered scheme (Wang et al., 2018).

According to this study, our efforts were dedicated to finding out the best optimization level of sterilants and immersion time by using non-linear MLP- NSGAII modeling and optimization procedure. In this way, making a strong link between the MLP model and NSGAII in our first priority in order to achieve the highest efficiency and the optimum concentrations of sterilants as well as immersion times during *in vitro* sterilization process. Generally, the objective of this study was to model and optimize the proper concentrations of sterilants and immersion times for sterilization of leaf explant of chrysanthemum, as a case study.

## Materials and Methods

### Case Study and Data

#### Plant Materials

The leaf explants of chrysanthemum “Hornbill Dark” were collected from grown greenhouse mother plants. The leaf explants were washed with tap water for 30 min and washed again after cleaning with a liquid soap solution. Additional surface sterilization was applied in a laminar airflow chamber. The explants were sterilized with 70% aqueous ethanol for 40 s, dipped into different concentrations and types of sterilants at various immersion times, and washed three times with sterilized distilled water. Afterward, 25 mm^2^ leaf segments (abaxial side) were incubated on 200-ml glass flasks containing 40 ml basal medium.

#### Media and Culture Condition

MS Murashige and Skoog (1962) medium as a basal medium used in this experiment having 0.7% agar (Duchefa Biochemie, Netherlands) and 3% sucrose. pH of the medium was adjusted to 5.8 using 1 N KOH or 1 N HCl before autoclaving at 121°C for 20 min. All cultures were kept at 26 ± 2°C under a 16-h photoperiod with light intensity of 50 μmol m^−2^s^−1^.

#### Experimental Design

The experiments were conducted based on completely randomized design (CRD) with a factorial arrangement with 15 replicates per treatment following with three sub-sets.

The effect of sterilants and immersion times on *in vitro* sterilization of chrysanthemum were evaluated based on the six following treatments;

1. Different concentrations of NaOCl (0, 0.5, 1, 1.5, and 2%) and immersion times (5, 10, and 15 min) effect on sterilization were evaluated (Table 1).
2. Different concentrations of Ca(ClO)_2_ (0, 8.5, 9, 9.5, and 10%) and immersion times (5, 10, and 15 min) effect on sterilization were evaluated (Table 2).
3. Different concentrations of HgCl_2_ (0, 0.25, 0.5, 0.75, and 1%) and immersion times (2.5, 5, and 7.5 min) effect on sterilization were evaluated (Table 3).
4. Different concentrations of H_2_O_2_ (0, 10.5, 11, 11.5, and 12%) and immersion times (5, 10, and 15 min) effect on sterilization were evaluated (Table 4).
5. Different concentrations of AgNO_3_ (0, 0.25, 0.5, 0.75, and 1%) and immersion times (5, 10, and 15 min) effect on sterilization were evaluated (Table 5).
6. Different concentrations of Nano-silver (0, 2.5, 5, 7.5, and 10 mg/L) and immersion times (5, 7.5, and 10 min) effect on sterilization were evaluated (Table 6).

**Table 1:** Effect of different concentrations of NaOCl at various immersion times on *in vitro* sterilization of chrysanthemum.

**Treatments**	**Contamination frequency (%)**	**Explant viability (%)**
**NaOCl (%)**		
0	100.00 ± 00.00	00.00 ± 00.00
0.5	44.44 ± 4.00	47.41 ± 5.38
1	23.70 ± 3.53	71.11 ± 3.33
1.5	6.67 ± 2.48	90.37 ± 3.53
2	2.96 ± 1.61	80.00 ± 3.51
**Time (min)**		
5	43.55 ± 8.85	51.55 ± 8.42
10	34.22 ± 9.76	58.22 ± 9.19
15	28.89 ± 10.02	63.55 ± 9.16
**NaOCl (%)** **×Time (min)**		
0 × 5	100.00 ± 0.00	0.00 ± 0.00
0 × 10	100.00 ± 0.00	0.00 ± 0.00
0 × 15	100.00 ± 0.00	0.00 ± 0.00
0.5 × 5	57.78 ± 2.22	33.33 ± 3.85
0.5 × 10	44.45 ± 2.22	42.22 ± 5.88
0.5 × 15	31.11 ± 2.22	66.67 ± 0.00
1 × 5	35.55 ± 2.22	62.22 ± 2.22
1 × 10	22.22 ± 2.22	71.11 ± 2.22
1 × 15	13.33 ± 3.85	80.00 ± 6.67
1.5 × 5	15.55 ± 2.22	77.78 ± 2.22
1.5 × 10	4.45 ± 2.22	93.33 ± 3.85
1.5 × 15	0.00 ± 0.00	100.00 ± 0.00
2 × 5	8.89 ± 2.22	84.44 ± 4.44
2 × 10	0.00 ± 0.00	84.44 ± 5.88
2 × 15	0.00 ± 0.00	71.11 ± 5.88

*Values in each column represent means ±SE*.

**Table 2:** Effect of different concentrations of Ca(ClO)_2_ at various immersion times on *in vitro* sterilization of chrysanthemum.

**Treatments**	**Contamination frequency (%)**	**Explant viability (%)**
**Ca(ClO)**_**2**_ **(%)**		
0	100.00 ± 00.00	00.00 ± 00.00
8.5	38.51 ± 3.10	57.04 ± 3.35
9	22.96 ± 4.17	71.11 ± 3.68
9.5	7.41 ± 2.59	88.89 ± 2.72
10	00.00 ± 00.00	88.15 ± 4.12
**Time (min)**		
5	40.44 ± 9.19	56.44 ± 8.97
10	32.89 ± 9.67	62.67 ± 9.04
15	28.00 ± 10.05	64.00 ± 9.06
**Ca(ClO)**_**2**_ **(%)** **×Time (min)**		
0 × 5	100.00 ± 00.00	00.00 ± 00.00
0 × 10	100.00 ± 00.00	00.00 ± 00.00
0 × 15	100.00 ± 00.00	00.00 ± 00.00
8.5 × 5	48.89 ± 2.22	46.67 ± 3.85
8.5 × 10	37.78 ± 2.22	57.78 ± 2.22
8.5 × 15	28.89 ± 2.22	66.67 ± 3.85
9 × 5	37.78 ± 2.22	57.78 ± 2.22
9 × 10	20.00 ± 3.85	73.34 ± 00.00
9 × 15	11.11 ± 2.22	82.22 ± 2.22
9.5 × 5	15.55 ± 2.22	80.00 ± 3.85
9.5 × 10	6.67 ± 3.85	91.11 ± 2.22
9.5 × 15	00.00 ± 00.00	95.56 ± 2.22
10 × 5	00.00 ± 00.00	97.78 ± 2.22
10 × 10	00.00 ± 00.00	91.11 ± 2.22
10 × 15	00.00 ± 00.00	75.56 ± 8.02

*Values in each column represent means ±SE*.

**Table 3:** Effect of different concentrations of HgCl_2_ at various immersion times on *in vitro* sterilization of chrysanthemum.

**Treatments**	**Contamination frequency (%)**	**Explant viability (%)**
**HgCl**_**2**_ **(%)**		
0	100.00 ± 00.00	00.00 ± 00.00
0.25	10.37 ± 2.25	57.04 ± 3.70
0.5	2.22 ± 1.11	42.22 ± 4.16
0.75	00.00 ± 00.00	12.59 ± 3.59
1	00.00 ± 00.00	2.22 ± 1.11
**Time (min)**		
2.5	24.00 ± 10.31	29.78 ± 7.10
5	22.67 ± 10.41	23.11 ± 6.76
7.5	20.88 ± 10.62	15.56 ± 4.86
**HgCl**_**2**_ **(%)** **×Time (min)**		
0 × 2.5	100.00 ± 00.00	00.00 ± 00.00
0 × 5	100.00 ± 00.00	00.00 ± 00.00
0 × 7.5	100.00 ± 00.00	00.00 ± 00.00
0.25 × 2.5	15.56 ± 4.45	64.44 ± 4.45
0.25 × 5	11.11 ± 2.22	62.22 ± 4.45
0.25 × 7.5	4.44 ± 2.22	44.44 ± 2.22
0.5 × 2.5	4.44 ± 2.22	55.56 ± 2.22
0.5 × 5	2.22 ± 2.22	42.22 ± 4.45
0.5 × 7.5	00.00 ± 00.00	28.89 ± 2.22
0.75 × 2.5	00.00 ± 00.00	24.44 ± 4.45
0.75 × 5	00.00 ± 00.00	8.89 ± 4.45
0.75 × 7.5	00.00 ± 00.00	4.44 ± 2.22
1 × 2.5	00.00 ± 00.00	4.44 ± 2.22
1 × 5	00.00 ± 00.00	2.22 ± 2.22
1 × 7.5	00.00 ± 00.00	00.00 ± 00.00

*Values in each column represent means ±SE*.

**Table 4:** Effect of different concentrations of H_2_O_2_ at various immersion times on *in vitro* sterilization of chrysanthemum.

**Treatments**	**Contamination frequency (%)**	**Explant viability (%)**
**H**_**2**_**O**_**2**_ **(%)**		
0	100.00 ± 00.00	00.00 ± 00.00
10.5	60.74 ± 2.82	37.78 ± 2.48
11	47.41 ± 2.34	49.63 ± 3.35
11.5	33.33 ± 4.30	62.22 ± 4.01
12	8.15 ± 2.43	90.37 ± 2.51
**Time (min)**		
5	56.44 ± 7.50	41.33 ± 7.28
10	50.67 ± 8.26	47.11 ± 8.06
15	42.67 ± 9.02	55.56 ± 8.80
**H**_**2**_**O**_**2**_ **(%)** **×Time (min)**		
0 × 5	100.00 ± 00.00	00.00 ± 00.00
0 × 10	100.00 ± 00.00	00.00 ± 00.00
0 × 15	100.00 ± 00.00	00.00 ± 00.00
10.5 × 5	68.89 ± 2.22	31.11 ± 2.22
10.5 × 10	62.22 ± 2.22	35.56 ± 2.22
10.5 × 15	51.11 ± 2.22	46.67 ± 0.00
11 × 5	53.33 ± 3.85	42.22 ± 5.88
11 × 10	46.67 ± 3.85	48.89 ± 5.88
11 × 15	42.22 ± 2.22	57.78 ± 2.22
11.5 × 5	44.44 ± 2.22	51.11 ± 4.45
11.5 × 10	37.78 ± 2.22	60.00 ± 00.00
11.5 × 15	17.78 ± 4.45	75.56 ± 4.45
12 × 5	15.56 ± 2.22	82.22 ± 2.22
12 × 10	6.67 ± 3.85	91.11 ± 2.22
12 × 15	2.22 ± 2.22	97.78 ± 2.22

*Values in each column represent means ±SE*.

**Table 5:** Effect of different concentrations of AgNO_3_ at various immersion times on *in vitro* sterilization of chrysanthemum.

**Treatments**	**Contamination frequency (%)**	**Explant viability (%)**
**AgNO**_**3**_ **(%)**		
0	100.00 ± 00.00	00.00 ± 00.00
0.25	82.22 ± 2.22	15.56 ± 2.48
0.5	71.85 ± 2.16	22.96 ± 1.17
0.75	62.22 ± 3.51	34.07 ± 3.59
1	49.63 ± 4.32	42.96 ± 4.73
**Time (min)**		
5	80.44 ± 3.59	18.22 ± 3.41
10	72.44 ± 4.91	22.22 ± 4.20
15	66.67 ± 5.79	28.89 ± 5.36
**AgNO**_**3**_ **(%)** **×Time (min)**		
0 × 5	100.00 ± 00.00	00.00 ± 00.00
0 × 10	100.00 ± 00.00	00.00 ± 00.00
0 × 15	100.00 ± 00.00	00.00 ± 00.00
0.25 × 5	88.89 ± 2.22	11.11 ± 2.22
0.25 × 10	82.22 ± 2.22	13.33 ± 3.85
0.25 × 15	75.56 ± 2.22	22.22 ± 4.45
0.5 × 5	77.78 ± 2.22	22.22 ± 2.22
0.5 × 10	71.11 ± 2.22	22.22 ± 2.22
0.5 × 15	66.67 ± 3.85	24.44 ± 2.22
0.75 × 5	71.11 ± 4.45	26.67 ± 6.68
0.75 × 10	62.22 ± 2.22	35.56 ± 4.45
0.75 × 15	53.33 ± 6.68	40.00 ± 6.68
1 × 5	64.44 ± 4.45	31.11 ± 5.88
1 × 10	46.67 ± 3.85	40.00 ± 6.68
1 × 15	37.78 ± 2.22	57.78 ± 2.22

*Values in each column represent means ±SE*.

**Table 6:** Effect of different concentrations of Nano-Silver at various immersion times on *in vitro* sterilization of chrysanthemum.

**Treatments**	**Contamination frequency (%)**	**Explant viability (%)**
**Nano-Silver (NS, mg/L)**		
0	100.00 ± 00.00	00.00 ± 00.00
2.5	71.11 ± 3.33	26.67 ± 3.14
5	57.78 ± 2.72	40.00 ± 2.22
7.5	45.93 ± 2.82	54.07 ± 2.82
10	22.96 ± 3.53	77.04 ± 3.53
**Time (min)**		
5	67.11 ± 6.02	32.44 ± 6.03
7.5	58.22 ± 7.38	40.89 ± 7.47
10	53.33 ± 7.57	45.33 ± 7.57
**NS (mg/L)** **×Time (min)**		
0 × 5	100.00 ± 00.00	00.00 ± 00.00
0 × 7.5	100.00 ± 00.00	00.00 ± 00.00
0 × 10	100.00 ± 00.00	00.00 ± 00.00
2.5 × 5	80.00 ± 6.68	17.78 ± 4.44
2.5 × 7.5	71.11 ± 2.22	26.67 ± 3.85
2.5 × 10	62.22 ± 2.22	35.56 ± 2.22
5 × 5	64.44 ± 4.44	35.56 ± 4.44
5 × 7.5	57.78 ± 2.22	40.00 ± 3.85
5 × 10	51.11 ± 4.44	44.44 ± 2.22
7.5 × 5	55.56 ± 4.45	44.44 ± 2.22
7.5 × 7.5	44.44 ± 2.22	55.56 ± 2.22
7.5 × 10	37.78 ± 2.22	62.22 ± 2.22
10 × 5	35.56 ± 2.22	64.44 ± 2.22
10 × 7.5	17.78 ± 4.44	82.22 ± 4.44
10 × 10	15.56 ± 2.22	84.44 ± 2.22

*Values in each column represent means ±SE*.

After 21 days of culture, the efficiency of different concentrations and types of sterilants, as well as immersion times on contamination frequency (CF) and explant viability (EV) were determined. The obtained data were used for modeling and optimization procedure by using MLP- NSGAII.

#### Multilayer Perceptron (MLP) Model

**Figure 1:**
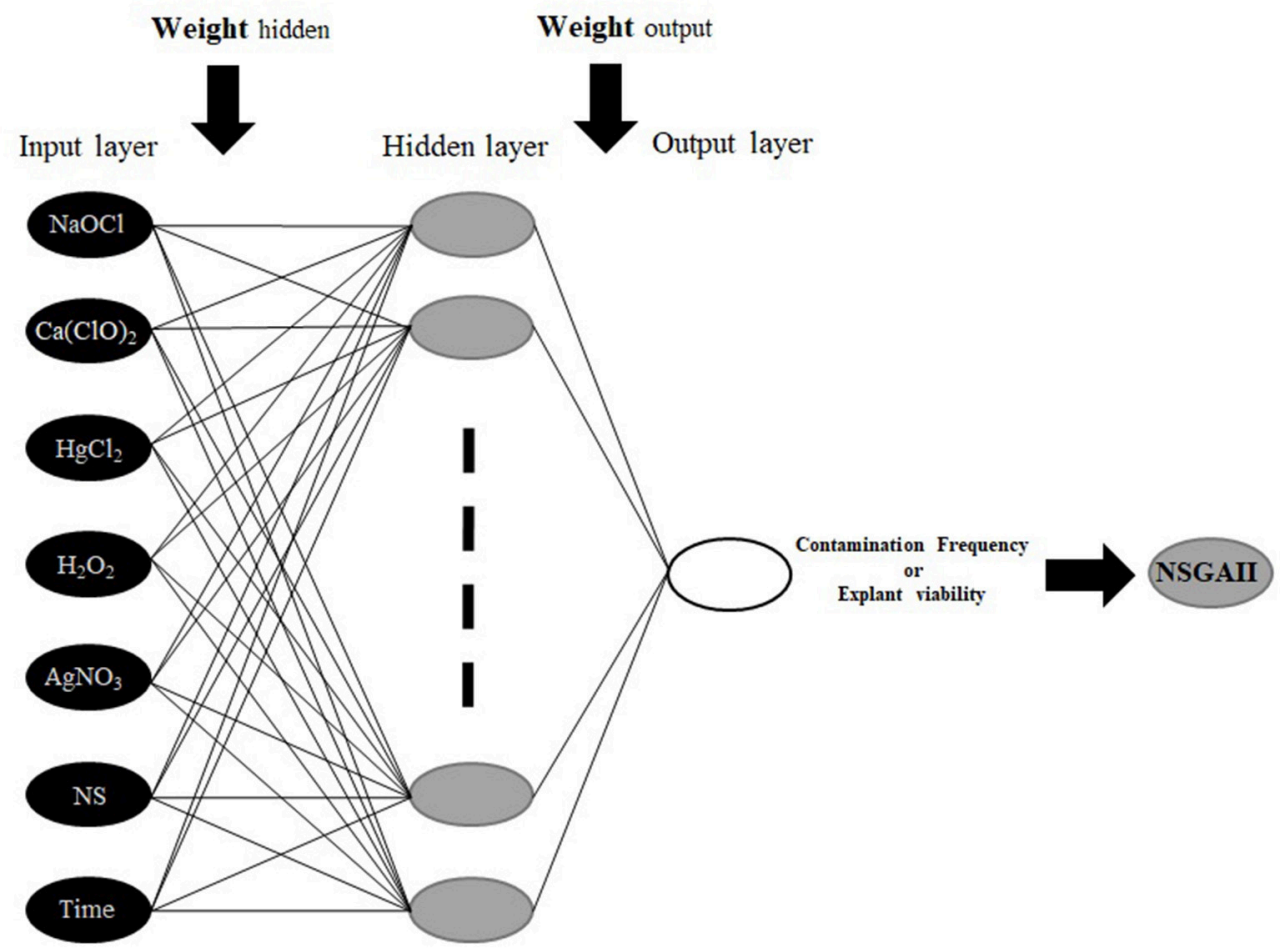
The schematic diagram of the proposed MLP methodology.

To construct MLP model; HgCl_2_, Ca(ClO)_2_, Nano-silver, H_2_O_2_, NaOCl, AgNO_3_, and immersion times were considered as inputs, and CF and EV were considered as outputs data for the modeling of *in vitro* sterilization (Figure 1). Also, 75 and 25% of the dataset were used to train and test the models, respectively. Moreover, the dataset was checked for confirming the range of train set contains the test data. To improve the performance of considered models and determine the best construct of each model, various values for significant model's parameters were tested based on a trial and error analysis. Finally, for each model, the best-resulted output with the minimum estimation error was determined based on Root Mean Square Error (RMSE) as well as the coefficient of determination (*R*^2^) as follows:

(1) $$R^{2}\;=\;1-\frac{\sum_{i=1}^{n}{\left(y_{i}-{\hat{y}}_{i}\right)}^{2}}{\sum_{i=1}^{n}{\left(y_{i}-{\bar{y}}_{i}\right)}^{2}}\;(0\leq R^{2}\leq 1)\;$$

(2) $$RMSE\;=\;\sqrt{\left(\sum_{i=1}^{n}{\left(y_{i}-{\hat{y}}_{i}\right)}^{2}\right)/n}\;(0\leq RMSE\leq +\infty )$$

Where n is the number of data, *y*_*i*_ is the value of predicted datasets, and ŷ_*i*_ is the value of observed datasets. Best fit can be indicated in the case that RMSE values closer to 0 and *R*^2^ values closer to 1.

The MLP is the most common types of ANN, consists of an input layer, one or more hidden layers, and an output layer (Hornik et al., 1989; Eslamian et al., 2009). MLP uses a supervised training procedure that consists of provided inputs and outputs to the network; the training process should be in such a way that the following function would be minimized:

(3) $$E=\frac{1}{K}\sum_{k=1}^{K}(y_{k}-{\hat{y}}_{k}{)}^{2}$$

Where *K is* the number of data, *y*_*k*_ is the *k*^*th*^ observation output, and ŷ_*k*_is the *k*^*th*^ predicted output. In a three-layer MLP with *m* neurons in the hidden layer and *n* input variables ŷ calculated as:

(4) $$\hat{y}=f\left[\sum_{j=1}^{m}w_{j}.g(\sum_{i=1}^{n}w_{ji}x_{i}+w_{j0})+w_{o}\right]$$

where *w*_*j*_: weight that connects of the *j*^*th*^ neuron of hidden layer and neuron of output layer, *w*_*ji*_: the weight connecting the *i*^*th*^ input variable and *j*^*th*^ neuron of hidden layer, *x*_*i*_: the *i*^*th*^input variable, *w*_*j*0_: bias of the *j*^*th*^ neuron of hidden layer, *w*_0_: bias related to the output neuron, *g*: the transfer functions for hidden layer, and *f:* transfer functions for the output layer.

Determining MLP architecture plays an important role in its efficiency (Khorsandi et al., 2011; Araghinejad et al., 2017). Therefore, in the architecture of an MLP, the number of hidden layers and the number of neurons in each layer should be determined. Hornik et al. (1989) showed that three-layer perceptrons with a sigmoid transfer function are universal approximators; which means that they can be trained to approximate any mapping between the inputs and outputs. Thus, the number of neurons in the hidden layer would be important in determining the architecture of an MLP. Some scholars have been suggested the appropriate number of neurons (m) based on a number of input (n) or the number of data (K). As an example, Tang and Fishwick (1993), Wong (1991), and Wanas et al. (1998) have offered “n,” “2n,” and “log (K)” as an appropriate number of neurons, respectively. Finally, by using trial and error method, the optimal number of neurons in the hidden layer should be determined while the reported offers can be used as a starting point. The low number of neurons makes the simplicity of the network and the large number of them makes the complexity of the network, therefore a simple network results in under-fitting, and vice versa.

In this study, feed forward back-propagation (3-layer back-propagation network), as the bases of the common network structure, was used for running an MLP model. For hidden and output layers transfer functions of hyperbolic tangent sigmoid (tansig) and linear (purelin) were applied, respectively. Also, for the training of the network, a Levenberg-Marquardt algorithm was applied for determining the optimal weights and bias.

#### Optimization Process (NSGA-II)

In order to select the best non-dominated solutions via a step-by-step procedure, NSGA-II should mainly depend on binary tournament selection, elitist non-dominated sorting, and crowding distance. The computational process should be started by initialization of the chromosome/population. Mutation operations, selection, and cross-over are three main components for simulation process that can be useful for evaluating objective functions and decision variables.

Afterward, the solutions, which are not dominated by the others and categorized as different non-dominated fronts of the population, are derived based on the non-dominated sorting concept. Each non-dominated front can be sorted as a rank or level data, and the population is ranked again except for the first Pareto front. Therefore, the non-dominated front, considered as the first rank, is the last generation of the optimal Pareto. The latest procedure is to remove the member that possess the highest rank (lower priority) and the select others to generate parent population of the next generation.

Afterward, each objective function should be estimated by crowding distance of a specific solution. Crowding distance is based on the average of two related neighboring solutions. Considering the lowest density of solutions that have less priority, the solutions of each level are categorized by crowding distance in descending order.

**Figure 2:**
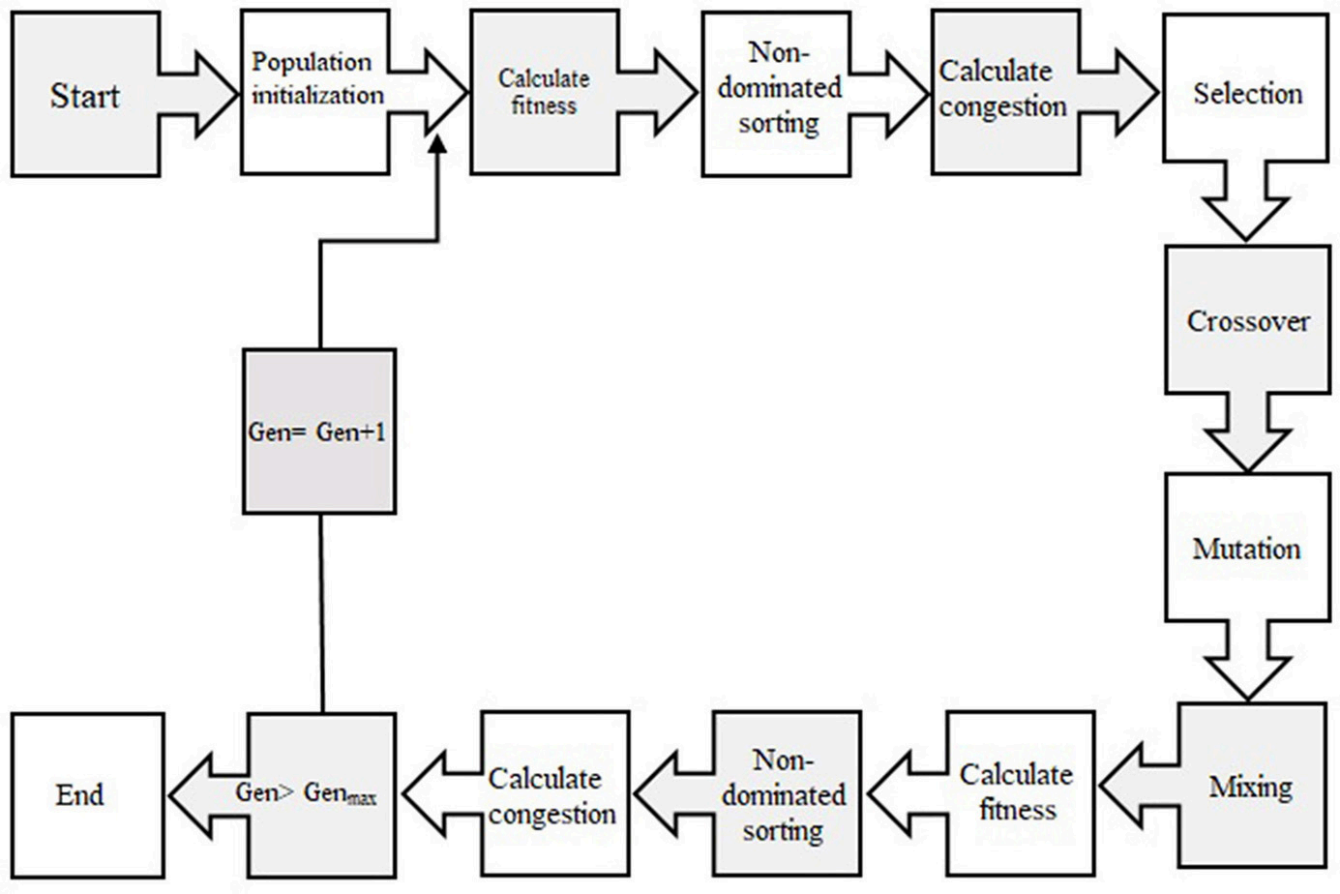
Schematic diagram showing the step-by-step NSGAII optimization process.

The next step after sorting solution is a selection step. The binary tournament selection operator is commonly used in the selection step. Therefore, a solution with greater crowding distance and lower rank will be chosen between two randomly solutions derived from the population/chromosome. Thus, children population is generated based on repeating the selection operator with applying the mutation operators and cross-over, same as the exact size of the parent population. Finally, the non-dominated sorting is utilized for the combination of children and parent populations after performing a simulation process for estimating the objective functions. The optimal solutions of each generation produce a new parent population during the last step “elitism” that final derived solution is known as the optimal Pareto front (Figure 2). In this study, CF and EV were considered as two objective functions to determine the optimum values of inputs. The ideal point of pareto was chosen such that CF and EV became the minimum and maximum, respectively. In other words, a point in the pareto front was considered as the solution such that

(5) $$\sqrt{{\left(CF-m\right)}^{2}+{\left(EV-n\right)}^{2}}$$

was minimal; where *m* and *n* are the minimum and the maximum CF and EV in observed data, respectively.

#### Sensitivity Analyses

The sensitivity CF and EV against the investigating growth elements was evaluated by using the following criterion;

The variable sensitivity error (VSE) value stands for the overall performance of the developed MLP model in the case that the particular independent variable is not available.

Variable sensitivity ratio (VSR) value: If all variables are available, VSR demonstrates the correlation between the error of the MLP model and VSE.

The higher the VSR, the more important variable will be. Therefore, all input variables can be ranked based on their importance.

The mathematical code was written conveniently for Matlab (version 9.5) software to construct and assess the models.

#### Validation Experiment

During validation experiment, the sterilants and immersion time optimized by MLP-NSGAII were tested for evaluating the efficiency of MLP-NSGAII to model and optimize the sterilants and immersion time for *in vitro* sterilization parameters (i.e., CF and EV).

## Results

(1) Effects of different NaOCl concentrations and immersion times on sterilization

Our results indicated that there was no contamination observed at 1.5% sodium hypochlorite for 15 min immersion time as well as 2% sodium hypochlorite for 10 and 15 min immersion times while the control (without NaOCl) treatments resulted in the highest CF (Table 1). Also, the highest EV (100%) was observed in 1.5% sodium hypochlorite for 15 min immersion time.

(2) Effects of various concentrations of Ca(ClO)_2_ and immersion times on sterilization

The highest EV (97.78%) was achieved in 10% Ca(ClO)_2_ for 5 min immersion time (Table 2). There was no contamination observed in 9.5% Ca(ClO)_2_ for 15 min immersion time as well as 10% Ca(ClO)_2_ for 5, 10, and 15 min immersion times. However, the highest CF (100%) was obtained at the control [without Ca(ClO)_2_] treatments.

(3) Effects of various concentrations of HgCl_2_ and immersion times on sterilization

No contamination was record for 0.5% HgCl_2_ for 7.5 min immersion time as well as 7.5% and 1% HgCl_2_ for 2.5, 5, and 7.5 min immersion times and the highest EV (55.56%) was observed in 0.5% HgCl_2_ for 2.5 min immersion time treatment. However, the lowest EV (0%) was achieved at the 1% HgCl_2_ for 7.5 min (Table 3).

(4) Effects of various concentrations of H_2_O_2_ and immersion times on sterilization

The highest (100%) and the lowest (2.22%) CF were found at the control (without H_2_O_2_) and 12% H_2_O_2_ for 15 min, respectively. Also, the highest EV (97.78%) was detected in 12% H_2_O_2_ for 15 min immersion time (Table 4).

(5) Effects of various concentrations of AgNO_3_ and immersion times on sterilization

The highest EV (57.78%) and the lowest CF (37.78%) was obtained at 1% AgNO_3_ for 15 min (Table 5).

(6) Effects of various concentrations of NS and immersion times on sterilization

The lowest CF (15.56%) along with the highest EV (84.44%) was obtained in 10 mg/L NS for 10 min immersion time (Table 6).

Based on these results, it would be clear that the type and concentration of sterilants along with explant exposure times to sterilant play a vital role in *in vitro* sterilization that each sterilant needs to be adjusted based on their optimum concentration and immersion time.

### MLP-NSGAII Modeling and Optimization

#### MLP Modeling and Evaluation

MLP was used for modeling the two outputs (CF and EV) based on seven variables including HgCl_2_, Ca(ClO)_2_, Nano-silver, H_2_O_2_, NaOCl, AgNO_3_, and immersion times.

**Figure 3:**
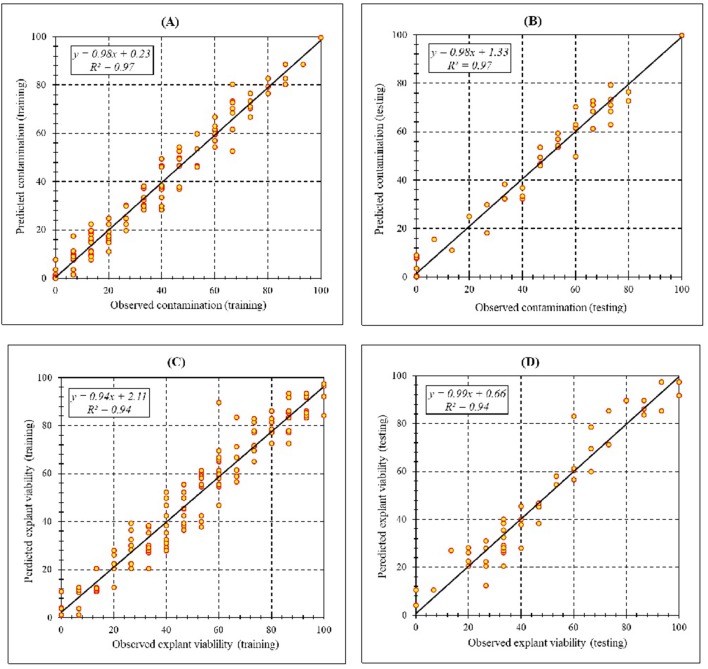
Scatter plot of model predicted vs. observed values of **(A)** Training set (*n* = 165); **(B)** testing set (*n* = 54) of contamination frequency, **(C)** training set (*n* = 165); **(D)** testing set (*n* = 54) of explant viability during *in vitro* sterilization of chrysanthemum obtained by MLP model. Fitted simple regression line on scatter points was indicated by a solid line.

Assessment of predicted and observed data describes the efficiency of the MLP model. According to Table 7, all of the *R*^2^ of training and testing data was over 94%. As can be seen in Table 7, the MLP model was successful in predicting CF and EV. Correlations between observed and predicted data for CF and EV demonstrated the good fit of the MLP model. The graphs (Figure 3) may apply to comprehend the perfect sterilization response and to measure the combined effects of sterilants and immersion times. The MLP model could precisely predict CF (*R*^2^ > 0.97), and EV (*R*^2^> 0.94) in the testing processes that were not used throughout the training data sets (Table 7). Furthermore, the trained MLP models of CF and EV had balanced performance criteria for both phases of training and testing. Generally, performance criteria (Table 7) illustrated that the MLP models were able to efficiently fit published data on the performances of *in vitro* sterilization to different types and concentrations of sterilants at different immersion times.

**Table 7:** Statistics of MLP models for contamination frequency (CF) and explant viability (EV) of chrysanthemum (training vs. testing values).

**Item**	**Contamination frequency**		**Explant viability**	
	**Training**	**Testing**	**Training**	**Testing**
R Square	0.97	0.97	0.94	0.94
RMSE	4.54	4.96	6.93	7.17

#### Model Optimization

##### The MLP-NSGAII predicted optimized various concentrations of sterilants at different immersion times

The ultimate purpose of this study was to analyze the MLP model to provide an accurate answer of what levels of sterilants and immersion times may be applied to obtain the maximum CF and EV. Thus, we have linked the model to NSGAII for finding the maximum efficiency and the optimum sterilants levels and immersion time which are essential for significant *in vitro* sterilization.

**Figure 4:**
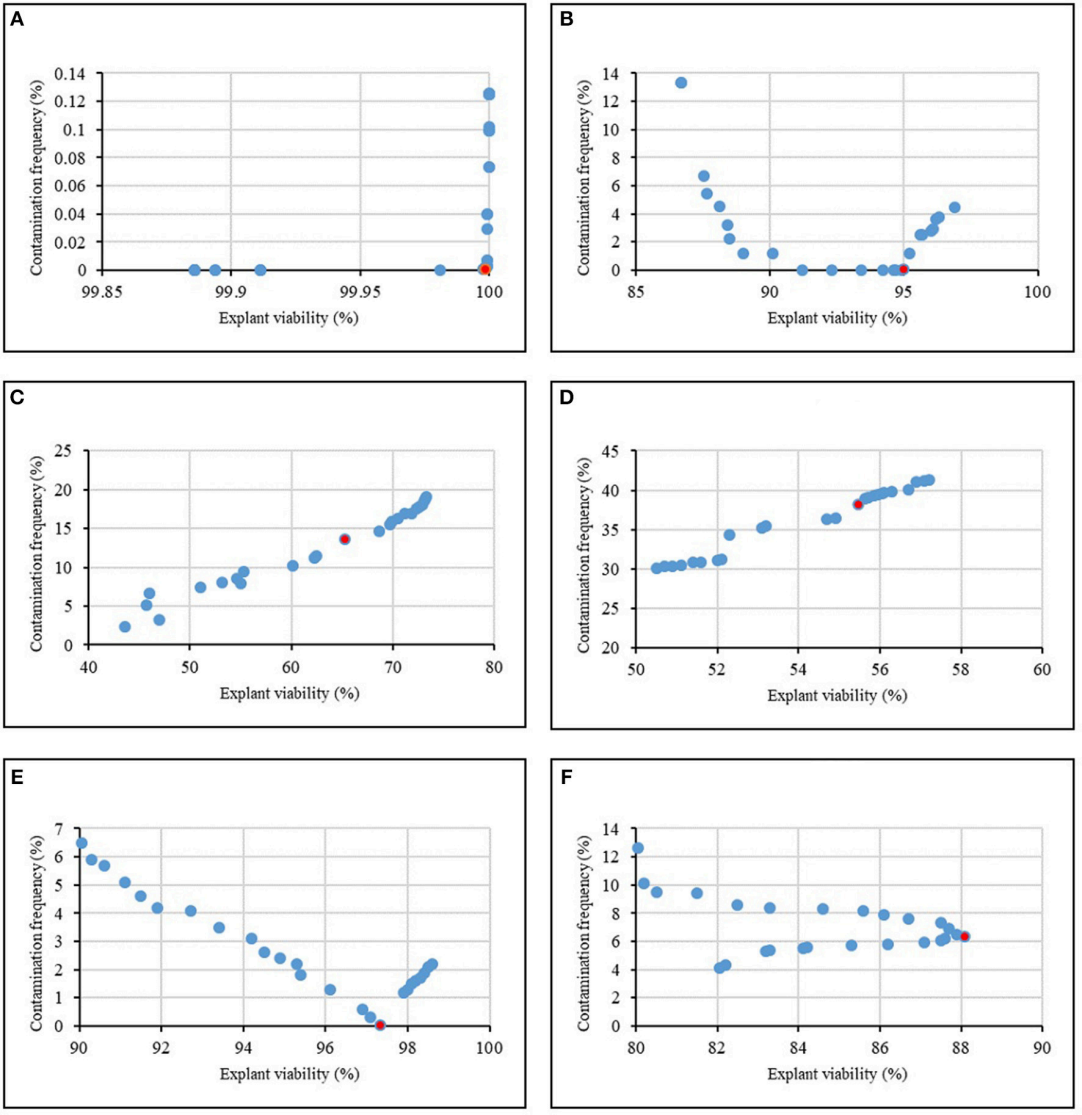
Pareto front obtained by NSGAII for the maximum explant viability and the minimum contamination frequency of chrysanthemum in various sterilants at different immersion times including **(A)** NaOCl + Time, **(B)** Ca(ClO)_2_ + Time, **(C)** HgCl_2_ + Time, **(D)** AgNO_3_ + Time, **(E)** H_2_O_2_ + Time, **(F)** Nano-Silver + Time. The red point indicates the ideal point.

Figure 4 and Table 8 showed the results of the optimization process. The lower bound and upper bound of input variables (Tables 1–6) were considered as constraints during the optimization process, and the point with the lowest CF and the highest EV was considered as the ideal point. As can be seen in Table 8, 1.62% NaOCl at 13.96 min immersion time caused 0% CF and 99.98% EV (Figure 4A), 10% Ca(ClO)_2_ at 7.43 min immersion time caused 0% CF and 95.01% EV (Figure 4B), 0.32% HgCl_2_ at 2.5 min immersion time caused 14.58% CF and 68.65% EV (Figure 4C), 0.92% AgNO_3_ at 14.5 min immersion time caused 38.17% CF and 55.49% EV (Figure 4D), 11.56% H_2_O_2_ at 15 min immersion time caused 0% CF and 97.34% EV (Figure 4E), and 10% NS at 15 min immersion time caused 6.31% CF and 88.10% EV (Figure 4F).

**Table 8:** Optimizing sterilants and immersion times according to optimization analysis on the developed MLP-NSGAII in the ideal point for contamination frequency (CF) and explant viability (EV) in chrysanthemum.

**Item**	**Input variable**							**Ideal point of EV**	**Ideal point of CF**
	**NaOCl (%)**	**Ca(ClO)_**2**_ (%)**	**HgCl_**2**_ (%)**	**AgNO_**3**_ (%)**	**H_**2**_O_**2**_ (%)**	**NS (mg/L)**	**Immersion times (min)**		
NaOCl + Time	1.62	0	0	0	0	0	13.96	99.98	0.00
Na(ClO)_2_ + Time	0	10.00	0	0	0	0	7.43	95.01	0.00
NgCl_2_ + Time	0	0	0.32	0	0	0	2.50	68.65	14.58
NgNO_3_ + Time	0	0	0	0.92	0	0	14.50	55.49	38.17
N_2_O_2_ + Time	0	0	0	0	11.56	0	15.00	97.34	0.00
NS + Time	0	0	0	0	0	10.00	11.63	88.10	6.31

In general, according to the MLP-NSGAII analysis results on different parameters of *in vitro* sterilization, NaOCl is predicted to be more proper than other sterilants in *in vitro* sterilization of leaf explants of chrysanthemum due to higher EV. Although the Ca(ClO)_2_ and HgCl_2_ resulted low CF, EV was lower than ones in NaOCl treatments.

##### Sensitivity analysis of the models

The comparative rank of input data was calculated through the entire 219 data lines (training and testing) to determine the general VSR. The VSR achieved for the model output (CF and EV), with respect to sterilants and immersion times (Table 9). Sensitivity analysis showed that CF was more sensitive to immersion time, followed by NaOCl, HgCl2, Ca(ClO)2, H2O2, NS, and AgNO3 (Table 9). In the EV model, the feed efficiency indicated more sensitivity for immersion time, followed by NaOCl, Ca(ClO)2, HgCl2, H2O2, NS, and AgNO3 (Table 9).

**Table 9:** Importance of inputs for contamination frequency (CF) and explant viability (EV) of chrysanthemum according to sensitivity analysis on the developed MLP model to rank the importance of inputs.

**Output**	**Item**	**NaOCl**	**Ca(ClO)_**2**_**	**HgCl_**2**_**	**AgNO_**3**_**	**H_**2**_O_**2**_**	**NS**	**Immersion times**
CF	VSR	3.25	2.28	3.11	1.12	1.89	1.71	3.48
	Rank	2	4	3	7	5	6	1
EV	VSR	2.34	2.12	1.63	1.35	1.56	1.50	2.49
	Rank	2	3	4	7	5	6	1

##### Validation experiment

The results of a validation experiment (Table 10) showed that MLP-NSGAII model could be able to specify the sterilants levels and immersion times for obtaining the most appropriate results for the studied parameters. The optimized sterilant levels and immersion time via MLP-NSGAII resulted in acceptable CF and EV which a little lower or higher than one predicted.

**Table 10:** Validation of the predicted data for contamination frequency (EF) and explant viability of chrysanthemum in validation experiment.

**Treatment**	**Contamination (%)**	**Explant viability (%)**
1.62% NaOCl × 13.96 min	00.00	100
10% Ca(ClO)_2_ × 7.43 min	00.00	95.55
0.32% HgCl_2_ × 2.5 min	13.33	62.22
11.56% H_2_O_2_ × 15 min	2.22	95.55
0.92% AgNO_3_ × 14.5 min	26.67	71.11
10 mg/L NS × 15 min	8.89	88.89

According to our results, MLP-NSGAII can be considered as one of the high applicable computational methods in analyzing data obtained of *in vitro* sterilization parameters for predicting optimized sterilants treatment (type and concentration of sterilants at different immersion times) required in the sterilization stage.

## Discussion

Being successful in plant tissue culture and releasing plant regeneration protocols are highly dependent on the efficiency of the sterilization stage (Da Silva et al., 2016a). This efficiency can be achieved through the optimized concentration of the sterilants as well as a period of exposure (Altan et al., 2010; Da Silva et al., 2016a; Hesami et al., 2018c). Although better sterilization can be obtained by the high concentration of sterilants with longer exposure, the explant viability can be negatively influenced by disinfectants at this condition, resulting in dehydrated-yellowish explant along with low viability (Da Silva et al., 2016a). Therefore, it is necessary to achieve the optimized level of disinfection which is appropriate for species—tissues and organs.

The desirable sterilization procedures should be proposed in a cheap, simple, efficient, and environmentally friendly way for eliminating the endogenous and surface contaminations (Purohit et al., 2011). In this study, we investigated the effects of various sterilants at different immersion times on *in vitro* contamination and explant viability of chrysanthemum, as a unique case study in this area via MLP-NSGAII.

High coefficient of determination between observed and predicted values for both training and testing process showed the accuracy of the models for the two parameters studied. The high efficiency of ANN in plant tissue culture has been shown by several studies (Gago et al., 2010b; Alanagh et al., 2014; Arab et al., 2016, 2018; Jamshidi et al., 2016; Nezami-Alanagh et al., 2017).

Our results showed that 1.5% NaOCl at 15 min immersion time resulted in 100% EV as well as no CF. Although there was no contamination at 2% NaOCl for 10 and 15 min immersion times, the EV was reduced. Therefore, our results confirmed that the EV could be reduced by increasing the concentration of NaOCl and immersion time. In accordance with our results, Hesami et al. (2018c) demonstrated that an increase in the concentration of NaOCl and immersion time had a negative effect on the explant viability of *Chenopodium quinoa*. In various studies in tissue culture of chrysanthemum, NaOCl was the most commonly utilized in the sterilization stage (May and Trigiano, 1991; Pavingerová et al., 1994; Tanaka et al., 2000; Da Silva, 2003; Shinoyama et al., 2004; Mandal and Datta, 2005; Xu et al., 2012; Naing et al., 2013). It is well established that NaOCl could be highly effective against various kinds of viruses, fungi, and bacteria (Da Silva et al., 2016a; Hesami et al., 2018c). Also, NaOCl is highly reactive with amides, nucleic acids, amines, and amino acids due to its strong oxidizing properties (Mihaljević et al., 2013). These reactions can produce the CO_2_, respective aldehyde, and NH_4_Cl (Da Silva et al., 2016b).

Ca(OCl)_2_ is known as a very effective sterilant with the poor water solubility (Boyette et al., 1993). Similar to the results of sodium hypochlorite effects, the explant viability was reduced by increasing the concentration of Ca(ClO)_2_ and immersion time. The positive effects of Ca(ClO)_2_ on *in vitro* sterilization have been shown in several studies in different species (Assareh and Sardabi, 2005; Mihaljević et al., 2013; Da Silva et al., 2016a).

Although 1% HgCl_2_ at different immersion times was caused to 0% CF, EV significantly decreased. The similarity to our results, Xu et al. (2005) showed that HgCl_2_ was better than NaClO and H_2_O_2_ for surface sterilization of *Pinellia ternata* (Thunb.) Breit. HgCl_2_ is generally believed to be a strong disinfectant (Marinescu et al., 2013). However, HgCl_2_ at high level causes to decrease in explant viability. Generally, the use of HgCl_2_ in sterilization stage is not recommended due to Hg neurotoxic and immunotoxic properties which are highly environmental pollutants (Marinescu et al., 2013; Da Silva et al., 2016b).

H_2_O_2_ is known as a non-phytotoxic chemical sterilizer that can be used for washing explants or added to the culture medium (even without autoclaving) (Curvetto et al., 2006). By the activity of catalases and peroxidases in the cell, H_2_O_2_ can be dissected into water and oxygen (Arora et al., 2002). This mechanism is known as a protection mechanism for preserving explant tissues from the adverse effect of H_2_O_2_ (Arora et al., 2002; Da Silva et al., 2016a). In our study, the treatment with 12% H_2_O_2_ at 15 min immersion time showed a low percent of contamination and a high percent of explant viability. Our findings are in agreement with the observation of Mihaljević et al. (2013) who reported that using H_2_O_2_ resulted in surface sterilization of sour cherry. Also, Curvetto et al. (2006) reported that an increase from 0.005 to 0.020% H_2_O_2_ reduced the contamination frequency from 52.5 to 40% in *Lilium longiflorum*. Also, Farooq et al. (2002) reported that using H_2_O_2_ resulted in 50% surface sterilization in *Annona squamosa* L.

In some cases, AgNO_3_ is used for extending the vase life of ornamental species such as geophytes (Da Silva et al., 2016a). Also, this solution can be useful in disinfection (Mihaljević et al., 2013), so the exogenous application might be induced floral development and initiation in shoot cultures and affected *in vitro* proliferation rate (Bais et al., 2000). The efficiency of AgNO_3_ as a disinfection solution can be limited due to the instability of AgNO_3_ in the presence of chemical components such as chlorides existed in soil-clinging organs or tap water (Newton et al., 1933). However, the effective results can be achieved in 0.05% AgNO_3_ supplemented with (0.15%) potassium cyanide (KCN; 1:3 w/w) (Newton et al., 1933). Our results showed that 1% AgNO_3_ at 15 min immersion time caused to 37.78% CF and 57.78% EV. Similar results were observed in *Trifolium pratense* L. by Campbell and Tomes (1984).

According to our results, 10 mg/L NS at 10 min immersion time caused to 15.56% CF and 84.44% EV. Similar results were reported by Arab et al. (2014) in *Prunus* rootstocks. The low concentration and exposure time (5–10 mg L^−1^ 5–10 min) of copper, gold, and silver nanocolloids have an antiviral, antifungal, and antibacterial activity that can be used as disinfection solution in plant tissue culture (Kim et al., 2017). Application of these solutions are free from washing explants with sterile distilled water (SDW) resulted in no damage to tissue (Da Silva et al., 2016a). Although the use of these disinfectants may increase recently, their influence on different plants and explants have not been studied yet.

Generally, NaOCl showed to be a better disinfectant agent in comparing with other sterilants which are in accordance with Campbell and Tomes (1984). They reported that NS, NaOCl, and Ca(ClO)_2_ were effective in reducing contamination of *Trifolium pratense* L. where NaOCl showed better performances. However, Assareh and Sardabi (2005) reported that among Ca(ClO)_2_, NaOCl, and HgCl_2_ for sterilization of *Ziziphus spina-christi* (L.) Desf. explants, 5% Ca(ClO)_2_ at 20 min was the most efficient.

## Conclusion

Plant tissue culture problems have to satisfy various conflict objective functions by considering different constraints. Therefore, there is a dire need of applying the multi-objective algorithm for the optimization process. This study has introduced MLP-NSGAII as a new computational tool for prediction-optimization of *in vitro* sterilization of chrysanthemum, as a case study. Based on the results, MLP-NSGAII could be able to identified interaction effects precisely and quickly rather than common statistical analysis for a large number of experiments. Finally, MLP-NSGAII can be recognized as a powerful method for utilizing in different areas of *in vitro* culture.

For further studies, it would be useful to evaluate and compare different multi-objective optimization algorithms in different areas of plant science, especially in plant tissue culture areas. Meanwhile, our results indicated that MLP-NSGAII can be recognized as a powerful method for utilizing in different areas of *in vitro* culture. Overall, MLP-NSGAII can be accelerated the development of efficient sterilization that addresses the needs of the future *in vitro* culture programs in different plants.

## Author Contributions

MH performed the experiments, data modeling, summed up, and wrote the manuscript. RN and MT designed and lead the experiments and revised the manuscript.

### Conflict of Interest Statement

The authors declare that the research was conducted in the absence of any commercial or financial relationships that could be construed as a potential conflict of interest.
